# Effect of Metformin Treatment on Serum Metabolic Profile Changes in Lean and Obese Zucker Rat Model for Fatty Liver Disease

**DOI:** 10.3390/biom13081234

**Published:** 2023-08-10

**Authors:** Stepan Melnyk, Reza Hakkak

**Affiliations:** 1 Arkansas Children’s Research Institute, Little Rock, AR 72202, USA; 2 Department of Dietetics and Nutrition, University of Arkansas for Medical Sciences, Little Rock, AR 72205, USA; 3 Department of Pediatrics, University of Arkansas for Medical Sciences, Little Rock, AR 72205, USA

**Keywords:** metformin, serum metabolic, obese rats

## Abstract

Excessive weight and obesity are the leading risk factors for the development of chronic diseases, including diabetes. Metformin is capable of significantly improving coexisting complications of diabetes. We used a metabolomics approach to examine the effects of metformin administration on lean and obese (fa/fa) Zucker rats. After 1 week of acclimation, twenty-eight 5-week-old female lean and obese rats were randomly assigned to and maintained in the following four groups (seven rats/group) for 10 weeks: (1) lean control (LC); (2) obese control (OC); (3) lean metformin (LM); and (4) obese metformin (OM). At the end of 10 weeks, serum was collected and analyzed using HPLC with electrochemical detection, HPLC with UV detection, and liquid chromatography mass spectrometry. We selected 50 metabolites’ peaks that were shared by all four groups of rats. Peak heights, as a defining factor, generally decreased in metformin-treated lean rats vs. untreated lean controls (3 LM:16 LC). Peak heights generally increased in metformin-treated obese rats vs. untreated obese controls (14 OM:5 OC). Overall, individual peaks were distributed as 11 that represented only lean rats, 11 that represented only obese rats, and 8 that were common among both lean and obese rats. In future studies, we will use a targeted metabolomics approach to identify those metabolites, map them to biochemical pathways and create a list of biomarkers. In summary, the current study contributed to a better understanding of the basic metabolic changes of lean and obese rats and demonstrated that both obesity and metformin make a significant impact on the metabolome of Zucker rats.

## 1. Introduction

Obesity is a multifactorial and complex chronic, relapsing, and even life-threatening condition characterized by an excessive accumulation of body fat. Since the 1970s, obesity prevalence in adults and children has increased globally, and currently, the number of obese or overweight people in the world is greater than the number of those who are underweight [1,2]. Excessive weight and obesity are the leading risk factors for the development of diabetes and other chronic diseases. Chronic sterile meta-inflammation accompanying obesity is the main contributor to both insulin resistance and the initiation of type 2 diabetes [3,4,5]. Adult type 2 diabetes has been considered a serious threat as it is frequently accompanied by several major complications, such as a higher prevalence of cancers, cardiovascular diseases, chronic kidney disease, osteoporosis, and neurodegenerative diseases. Thus, the increase in obesity prevalence has led to increases in diabetes and other comorbid conditions, along with a related increase in community health care costs and patient mortality [6,7].

**Figure 1:**
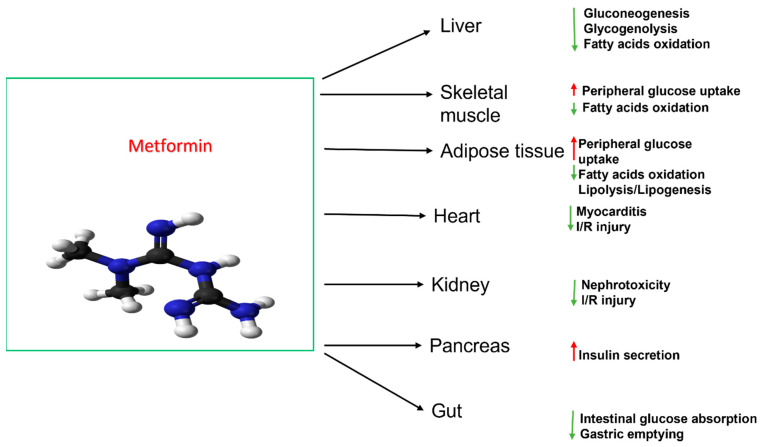
Metformin mechanism of action. (green color means decreasing risk and red is increasing risk).

One of the oldest and most widely used oral antidiabetic drugs is metformin, which is capable of significantly improving coexisting complications of diabetes [8]. Metformin is used as a first-line treatment for type 2 diabetes and is a natural cationic biguanide capable of utilizing a broad spectrum of molecular mechanisms to control blood glucose. These mechanisms range from decreasing intestinal glucose absorption and inhibition of hepatic gluconeogenesis to decreasing insulin resistance, although the exact details of these mechanisms are unknown [9]. The mechanism action of metformin is shown in Figure 1. 

Small molecules and their metabolites create unique and distinctive metabolic profiles for a biological system known as metabolome. The large-scale study of these small biomolecules are called metabolomics. Utilizing advanced technological tools, metabolomics are capable of not only performing a deep metabolic analysis of biological systems (human, animal, cell) at certain time points and under certain conditions (diseases, treatment), but are also able to provide a personalized approach. In a recent review by Costanzo et al. [10], the authors analyzed numerous human studies and concluded that metabolic differences in age and gender need to be adequately considered by investigators in future studies.

The use of a metabolomics approach could be extremely beneficial in the search for explanations on the multiple positive effects of metformin treatment. Metabolomic analyses have been applied to investigate metabolic alterations in response to metformin treatment and have shown significant systemic metabolome changes in a variety of biofluids, tissues, and cells [11]. The meta-analysis review by Xie et al. [12] systematized metabolic data from numerous studies that used a Zucker rat model to explain the development of obesity. The effect of metformin treatment on Zucker rats and the systemic changes of metabolism still need to be studied. Treatment with metformin in patients with non-alcoholic fatty liver disease (NAFLD) has been reported with contradictory findings regarding its effects on blood lipids and liver histology [13,14]. Obesity increases non-alcoholic fatty liver disease (NAFLD), and we recently reported that metformin treatment could reduce NAFLD using the obese Zucker rat model [15]. Using the same model, it was also observed that metformin treatment could change the gut microbiome [16]. 

Zucker rats (*fa*/*fa*) are the best-known, most widely used rat model for obesity and have been used to study non-insulin-dependent diabetes mellitus. The *fa* mutation, discovered by Zucker and Zucker in 1961, is a leptin receptor mutation that impairs satiety, leading to hyperphagia and obesity [17,18,19,20]. The Zucker rat is used as a model of human early onset hyperplastic–hypertrophic obesity. Obese Zucker rats develop hyperleptinemia, obesity, hyperinsulinemia, hyperlipidemia, insulin resistance, and hyperglycemia—classic signs of human metabolic syndrome [21]. As such, the obese Zucker rat may represent a useful tool to further understand the etiopathology of metabolic syndrome in humans. 

We recently reported that obesity significantly changed the metabolic profile of 62% of the selected metabolites in obese Zucker rats. In general, we observed metabolite increases in obese rats, compared to lean rats, that were randomly distributed across the serum metabolic profile, except for one segment where the level of metabolites in obese rats was consistently lower [22].

In the present study, we used metabolomics tools to better understand the fundamental metabolic consequences of metformin treatment on lean and obese Zucker rats. An evidence-based scientific approach to modulate and study this process will be helpful in further clinical studies.

## 2. Materials and Methods

Most of the details regarding experimental design, sample preparation, data collection, and analysis have been described previously [22]. We will present an abbreviated description of these steps. 

### 2.1. Experimental Design

Twenty-eight 5-week-old female lean and obese (*fa*/*fa*) Zucker rats (Envigo, Indianapolis, IN, USA) were used in this study. All animal care and procedures were approved by the University of Arkansas for Medical Sciences/Arkansas Children’s Research Institute Institutional Animal Care and Use Committee (protocol # 3882) and adhered to the institutional policies and procedures.

Animals were maintained according to USDA and NIH guidelines. After a 1-week acclimation, lean and obese rats were randomly assigned to the following four groups (7 rats/group) and maintained for 10 weeks: (1) lean control (LC), (2) obese control (OC), (3) lean administered metformin (LM), and (4) obese administered metformin (OM). After 10 weeks, all rats were euthanized and were sacrificed using CO_2_ (30%) prior to decapitation; serum was collected and stored at −80 °C for further analysis. 

### 2.2. Sample Preparation for Measurement of Serum Metabolites

Serum samples for chromatographic analyses were prepared by adding 100 μL of ice-cold 10% meta-phosphoric acid to 100 μL of serum followed by a 10 min incubation on ice, centrifugation (18,000× *g* for 15 min at 4 °C), and filtration (0.2 μm nylon filter). Aliquots of the resultant extract (20 μL) were analyzed with HPLC with electrochemical detection (ECD) and HPLC with ultraviolet detection (UV) (see below). 

For liquid chromatography mass spectrometry analyses (LC-MS), 50 μL of serum was mixed with 300 μL of HPLC-grade methanol, followed by centrifugation (18,000× *g* for 15 min at 4 °C). Supernatants were dried under nitrogen flow and then dissolved in 50 μL of 50% methanol/0.2% formic acid, after which 5 μL samples were injected and analyzed with LC-MS (see below).

### 2.3. HPLC and LC-MS Methods

#### 2.3.1. HPLC-ECD

Unique methodological details for metabolite analysis with HPLC-ECD have been published previously [23,24]. Serum extract samples (20 μL) were analyzed using an HPLC-ECD system (Model 580, ESA Inc., Chelmsford, MA, USA) with a Phenomenex (Phenomenex Inc., Torrance, CA, USA) Capcell Pak reverse phase C_18_ column (4.6 × 150 mm, 3 μm particle size). The isocratic mobile phase was composed of 50 mM sodium phosphate, 1.0 mM octanesulfonic acid, and 2% acetonitrile (*v*/*v*), adjusted to pH 2.7, at a flow rate of 1 mL/min and a column compartment thermostat temperature of 25 °C. Metabolite identification (based on retention time) and quantification (based on peak areas of the standard and sample) were performed using HPLC software (Dionex Corp., Sunnyvale, CA, USA, 7.3).

#### 2.3.2. HPLC-UV 

Serum extract samples (20 μL) were also analyzed using an HPLC-UV system (Thermo Scientific UltiMate 3000, Thermo Fisher Scientific Inc., Waltham, MA, USA) with a Raptor AR C_18_ column (150 × 4.6 mm, 2.7 μm particle size) purchased from Restek Co., (Bellefonte, PA, USA). The isocratic mobile phase was composed of 0.05 mM KH_2_PO_4_ and 10% acetonitrile (*v*/*v*) at a flow rate of 1 mL/min and UV detection at 220, 240, 260, and 280 nm. Peak identification and plasma concentration of metabolites, calculated from peak areas and standard calibration curves, were determined using HPLC software (Dionex Corp., Sunnyvale, CA, USA, 7.3).

#### 2.3.3. LC-MS 

LC-MS was performed with a Thermo Fisher Scientific LTQ XL mass spectrometer coupled with a Thermo Scientific UltiMate 3000 HPLC system with a +3.5 kV spray voltage, a 275 °C capillary temperature, a 300 °C heater temperature, a sheath gas flow of 25 L/min, an auxiliary gas flow of 10 L/min, and a mass scan range of 80–900 *m*/*z*. The mobile phase was a 30 min gradient flow of (A) 10 mM ammonium formate in 95:5 acetonitrile/water with 0.1% formic acid and (B) 10 mM ammonium formate in 50:50 acetonitrile/water with 0.1% formic acid at a flow rate of 300 µL/min. Extract samples (5 μL) were injected into a Thermo Fisher Scientific Accurore C_18_ column (100 × 2.1 mm, 2.6 μm particle size) with a column temperature of 40 °C. MS data collection and analysis were performed using Thermo Fisher Scientific Xcalibur™ software v 2.2 SP1.48. Metabolites and their concentrations were determined based on retention time and molecular weight (*m*/*z*) using Thermo Fisher Scientific Mass Frontier 7.0 software.

### 2.4. Data Processing and Statistical Analysis

Mass Frontier 7.0 (Thermo Fisher Scientific software, San Jose, CA, USA) was used for the management, evaluation, and interpretation of mass spectra. For statistical analyses, one-way ANOVA and SigmaPlot 13.0 software (Systat Software, Inc., San Jose, CA, USA) were used, and the results are presented as mean ± standard deviation (SD). Student’s two-tailed *t* test was used for independent comparisons of the heights of common peaks between the groups of Zucker rats. A *p*-value < 0.05 was considered a significant difference.

## 3. Results

### 3.1. Body Weight 

The mean ± SD body weight (g) of lean and obese rats at the beginning of the experiment were as follows: lean (*n* = 14), 98 ± 7 g; obese (*n* = 14), 155 ± 13 g. After 10 weeks, the body weight of untreated lean and obese rats and those treated with metformin were LC, 269 ± 26 g; OC, 590 ± 41; LM, 278 ± 17 g; and OM, 573 ± 48 g. Our results showed that obese rats gained significantly (<0.001) more weight than lean rats after 8 weeks. Also, after 10 weeks of metformin treatment, obese rats gained body weight significantly higher in both the control and metformin treatment groups (*p* < 0.001) compared to lean rats. However, there were no differences (*p* = 0.20) between OC and OM groups. 

### 3.2. Untargeted Metabolomics Data

To generate the data for this study, we used an untargeted metabolomics approach using different analytical liquid chromatographic platforms (HPLC-ECD, HPLC-UV, and LC-MS). We were able to collect over 400 high-quality (clean and distinguished peaks on the chromatograms) and high-intensity (the minimum noise-to-peak ratio was 1:10) peaks. We selected the 50 highest peaks that were shared between the four groups of rats. Each peak represented a single metabolite.

**Figure 2:**
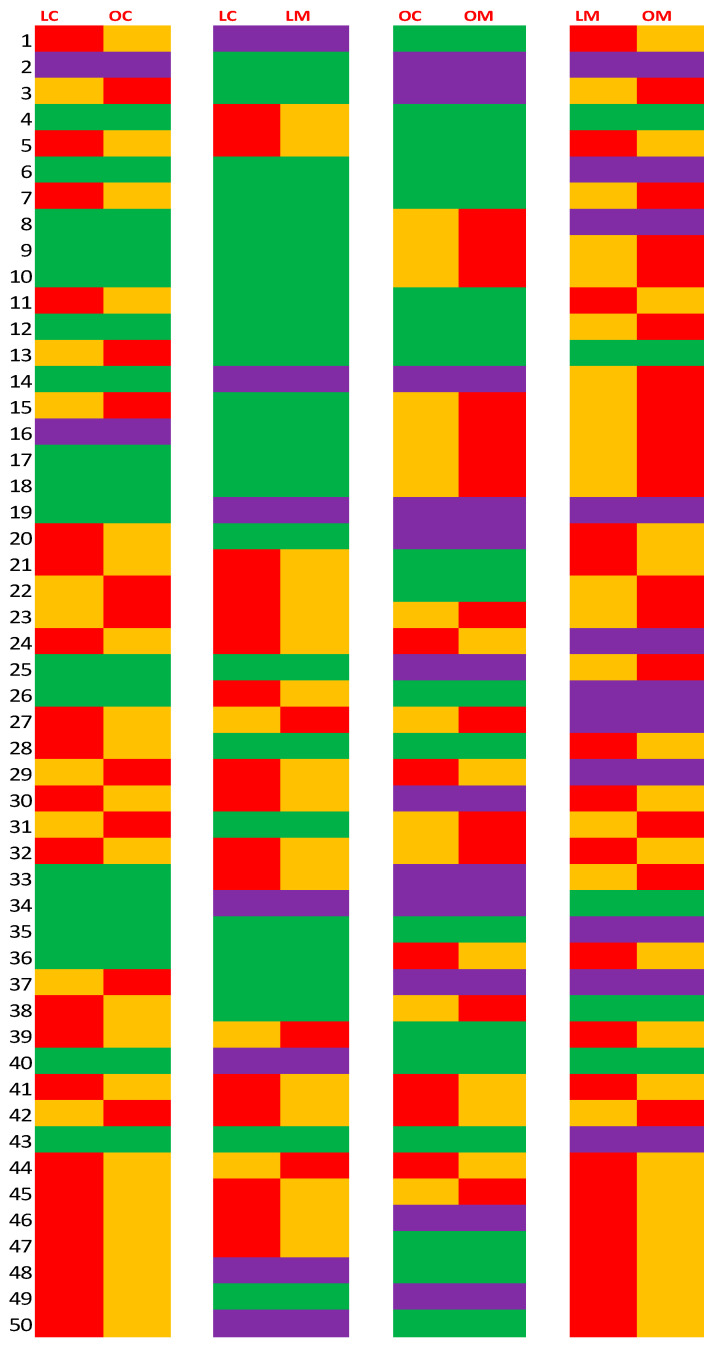
Heat map of metabolic differences in serum between lean and obese, control and metformin-treated Zucker rats. 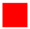 Statistically higher (*p* < 0.05); 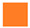 Statistically lower (*p* < 0.05); 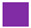 Marginal (0.1 > *p* > 0.05); 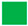 No difference (*p* > 0.05).

Analysis of all 50 individual peaks and average peak heights of seven LC or OC and seven LM or OM rats are presented in Figure 2. Student’s *t* test was used to compare the average heights between the groups. We divided these peaks into two different categories: (1) groups not significantly different (*p* > 0.05) with subcategory of slight difference between the groups with *p*-values between 0.1 and 0.05; and (2) groups significantly different (*p* < 0.05).

**Figure 3:**
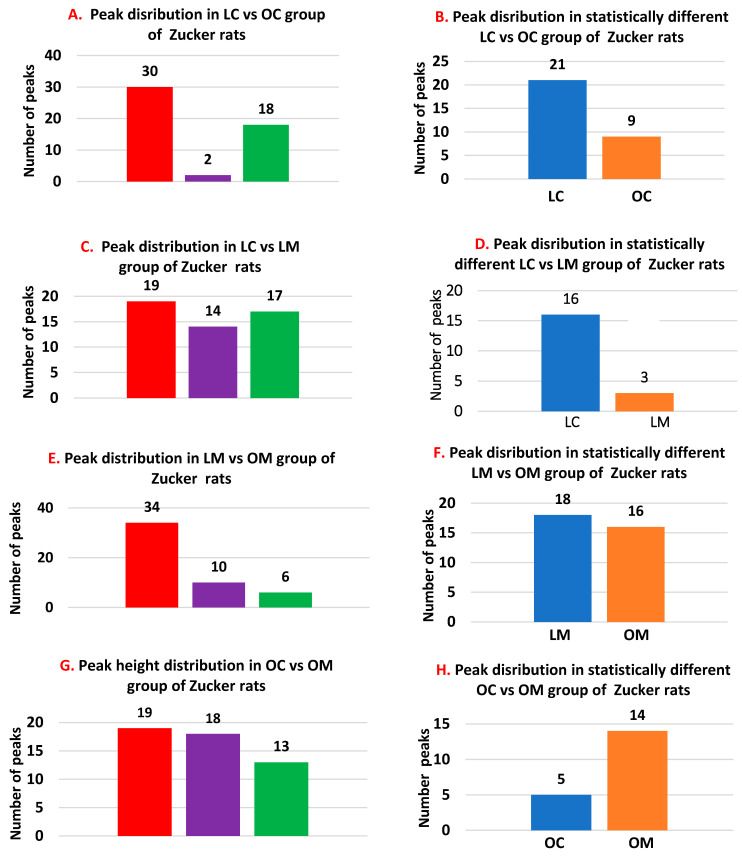
Peaks distribution in lean control (LC), lean metformin (LM), obese control (OC), and obese metformin (OM) rats. 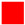 *p* < 0.05, 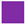 0.1 > *p* > 0.05, 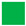 *p* > 0.05.

Based on our data in Figure 2, we compared the metabolic profiles of the LC and OC rats and found that 30 peaks (Figure 3A) demonstrated statistically different heights, 18 peaks demonstrated no differences, and 2 peaks demonstrated marginal differences. Statistically different groups were further divided (Figure 3B) into two subgroups: 21 peaks were higher in LC rats, and only 9 peaks were higher in OC rats.

By comparing the peak heights of the LC and LM groups (Figure 3C), we found that 19 peaks demonstrated statistical differences, 17 peaks demonstrated no differences, and 14 peaks demonstrated marginal differences. Statistically different groups were further divided (Figure 3D) into two subgroups: 16 peaks were higher in LC rats, and only 3 peaks were higher in LM rats.

Upon comparing the peak heights of the LM and OM groups (Figure 3E), we found that 34 peaks demonstrated statistical differences, 6 peaks demonstrated no differences, and 10 peaks demonstrated marginal differences. Statistically different groups were further divided (Figure 3F) into two subgroups: 18 peaks were higher in LM rats, and 16 peaks were higher in OM rats. 

Finally, by comparing the peak heights of the OC and OM groups (Figure 3G), we found that 19 peaks demonstrated statistical difference, 13 peaks demonstrated no difference, and 18 peaks demonstrated marginal differences between animals. Statistically different groups were further divided (Figure 3H) into two subgroups: only 5 peaks were higher in OC rats, and 14 peaks were higher in OM rats.

**Figure 4:**
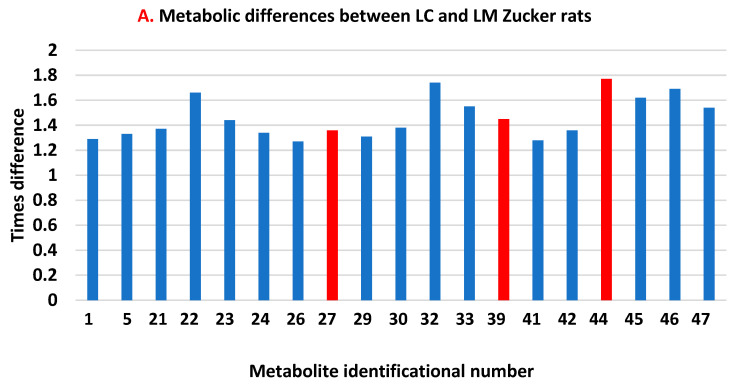

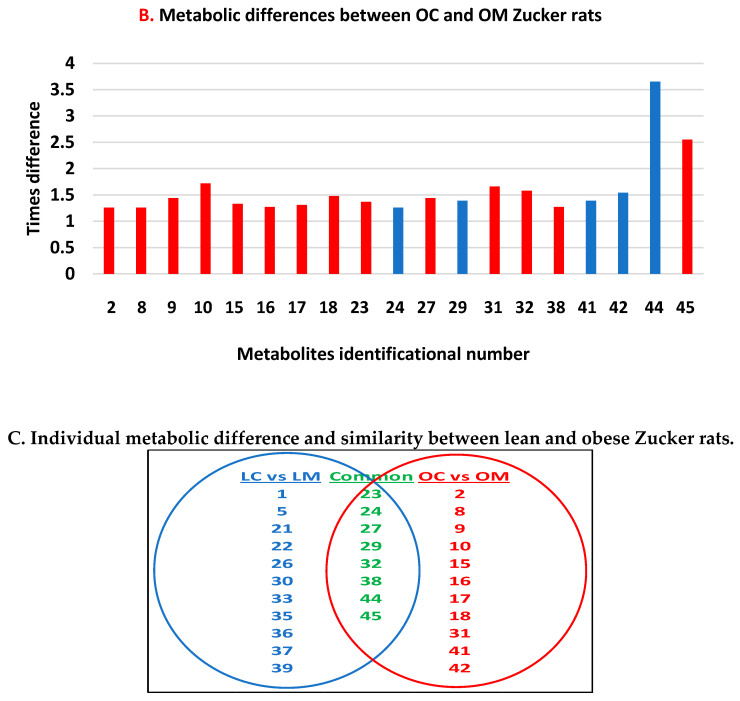
Metabolic differences in individual peak heights between lean and obese Zucker rats. 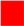 Peaks more pronounced in obese animals. 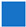 Peaks more pronounced in lean animals. 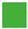 Peak that are similar among lean and obese animals.

After calculating the total number of peaks in the statistically higher groups of Zucker rats, we analyzed their distribution according to the individual number (ID) of peaks between groups of animals. We observed that the metformin treatment of lean rats (LC vs. LM) (Figure 4A) demonstrated a predominant (16:3) decrease in the height of the peaks, and the metformin treatment of obese animals (OC vs. OM) (Figure 4B) demonstrated a predominant (14:5) increase in the height of the peaks. All individual peaks could be divided into three subcategories: (1) individual higher peak IDs that represent only lean animals (LC vs. LM), (2) individual higher peak IDs that represent only obese animals (OC vs. OM), and (3) individual higher peak IDs that are common between the two groups (Figure 4C).

## 4. Discussion 

We used a lean and obese Zucker rat model to investigate the effects of metformin treatment on serum metabolic changes using an untargeted metabolomics approach. The studies of metabolic reactions in Zucker rats are very important for the generation of basic systemic knowledge of metabolism and to direct further steps in experimentation with this rat model. Few investigators have reported [25] data regarding metabolic changes in Zucker rats that were collected using a metabolomics approach [26]. The majority of research focuses on a better understanding of metformin’s protective mechanisms related to certain pathological conditions, including diabetes, but does not focus on fundamental metabolic changes [27,28,29,30,31] or even extend applications of metformin [32]. The general metabolic studies of the effect of metformin on Zucker rats that have used a metabolomics approach have been extremely limited. 

In the present study, we introduced a combination of analytical platforms from those that are complicated and technically demanding, such as LC-MS, to the relatively simple and affordable ones, such as HPLC-UV and HPLC-ECD. Our results demonstrated excellent practical potential and advantages whether we used all platforms together or separately in metabolomics studies. 

Previously, we reported that the basic metabolic status of untreated lean and obese Zucker rats (LC and OC in this study) demonstrated a difference in 60% of the metabolites we analyzed and also demonstrated significant differences in peak heights (amplitude) and direction (shift of the peaks toward lean or obese group) [22]. These differences were present in both lean and obese animals, but the height of the selected peaks’ domination (42% vs. 18%) was present in lean animals compared to obese. Such basic phenotypic differences could also predict metabolism. These metabolites could be critical in the use of targeted metabolomics and identification in future experiments in Zucker rats and other models. Our data demonstrated that the treatment of Zucker rats with metformin led to a variety of changes in metabolic status in both lean and obese animals. These metabolic changes/modifications, however, developed a different amplitude and direction in lean and obese animals. We found that peak heights (which represent metabolite concentrations) in lean control and lean metformin-treated rats were affected in approximately one-third of metabolites of interest with definite domination in the lean control group compared to the metformin-treated group (84% in LC vs. 16% in LM). On the other hand, metformin treatment resulted in decreased serum concentrations of certain metabolites in lean rats. In obese rats, we observed similar total metabolic changes (38%) in peak amplitudes but in the opposite direction (31% in OC vs. 69% in OM). The metformin treatment of obese rats stimulated an increase in some metabolite concentrations. 

**Figure 5:**
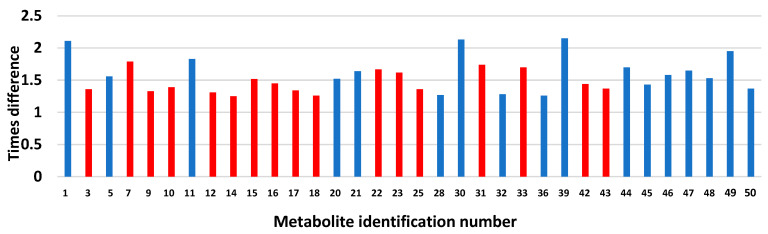
Changes of individual peak heights of metformin-treated lean and obese Zucker rats. 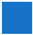 Peaks higher in the lean group. 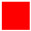 Peaks higher in the obese group.

The different directions in the reaction of metformin-treated lean and obese rats make us question how each individual metabolite (from the 50 selected) will react in lean and obese rats. We found that lean and obese rats have a pool of eight common metabolites that react in the same direction in both groups and a pool of 11 distinct metabolites for each group. These 11 metabolites in obese rats could potentially be good candidates for further study by targeted metabolomics to identify, quantify, and tie them to specific metabolic pathways. They could also be good candidates for the determination of treatment biomarkers. In general, upon comparing peak heights of lean and obese metformin-treated (LM vs. OM) rat groups (Figure 5), we observed that 68% of peaks/metabolites affected were statistically different. They were distributed equally (17 in LM and 17 in OM), and each animal group had its own pool of peaks/metabolites that independently reacted to the metformin treatment. 

The protective effect of metformin alone or in combination with other medical treatment of diabetes have been reported in human and animal model studies. This effect is very complex, multifactorial, and most importantly, not fully understood. In our current study, we applied an untargeted metabolomics approach to maximize the data collection from our analytical installation of an obese Zucker rat model. These data will contribute to better understanding the metabolic bases of metformin effects on obese animals with promise for a future better manipulation through diet/treatment to optimize the protective effect of metformin. We found (Figure 4B) that 19 out of 50 (38%) of the metabolites in obese Zucker rats react to the treatment of metformin by increasing/decreasing their levels in serum. The identification of these metabolites is a critically important future step of our research.

Previously, as was reported in [22], there is a significant difference in serum metabolic profile between lean and obese Zucker rats. We reported that obese rats had a significantly lower methionine, free cysteine, tryptophan, kynurenic acid, and higher levels of cystine and tyrosine compared to lean rats. Cysteine/cystine ratio, also known as an oxidative ratio [33], was lower in obese rats. This couple plays an important reductive/oxidative role in support of oxidative homeostasis, and along with the decrease in the ratio, obese rats are becoming more vulnerable to oxidative stress changes and damages. In the present study, we selected the same metabolic row for a comparison in obese control and obese metformin-treated Zucker rats (Table 1). We noticed that the metformin treatment of obese rats is also capable of changing a metabolic profile in its own unique way. It was observed to increase the levels of free cysteine, tryptophan, and kynurenic acid in obese metformin-treated animals compared to control animals. Such changes can positively affect resistance to oxidative stress through the further involvement of cysteine in the synthesis of glutathione as well as affect the gut–brain axis through the involvement of tryptophan and kynurenic acid. The question about a full scale of metabolic perturbations, including the interconnection between these two pathways mentioned above, are open for further study.

## 5. Conclusions

The current study helped us to better understand the basic metabolic changes in lean and obese Zucker rats with and without metformin treatment. Our data showed that both obesity and metformin treatment make significant impacts on the metabolome of Zucker rats, including oxidative stress and the gut–brain axis metabolites and their mutual interactions. These observed changes need to be further explored with a much more detailed targeted metabolomics approach to discover metabolic biomarkers.

Such biomarkers could be extremely helpful in the clarification of our options for choosing adequate tools for assessing the metabolic status of experimental obesity models using an untargeted metabolomics approach. In addition, biomarkers discovered in the future will help to identify the involvement of biochemical pathways in the treatment and management of obesity and glucose-lowering actions.

## Figures and Tables

**Table 1:** Serum metabolite concentrations of obese control (OC) and obese metformin (OM)-treated rats.

	OC	OM	*p*-Value
Methionine, nmol/mL	36.4 ± 5.72	34.4 ± 10.18	0.34
Cysteine, nmol/mL	10.1 ± 1.34	12.74 ± 2.61	0.024
Cystine, nmol/mL	14.7 ± 2.71	18.69 ± 5.75	0.071
Cysteine/Cystine	0.71 ± 0.168	0.74 ± 0.317	0.42
Tryptophan, nmol/mL	39.1 ± 9.14	48.2 ± 6.28	0.029
Kynurenic acid, nmol/mL	1.66 ± 0.332	2.27 ± 0.576	0.023
Tryptophane/Kynurenic acid	24.6 ± 8.02	22.7 ± 7.04	0.32
Tyrosine, nmol/mL	62.2 ± 10.83	66.5 ± 8.21	0.21

## Data Availability

The data presented in this study are available in this article.
